# Reducing the Decline in Physical Activity during Pregnancy: A Systematic Review of Behaviour Change Interventions

**DOI:** 10.1371/journal.pone.0066385

**Published:** 2013-06-14

**Authors:** Sinead Currie, Marlene Sinclair, Marie H. Murphy, Elaine Madden, Lynn Dunwoody, Dianne Liddle

**Affiliations:** 1 Maternal, Fetal and Infant Research Centre, Institute of Nursing and Health Research, University of Ulster, Newtownabbey, Northern Ireland; 2 Centre for Physical Activity and Health Research, Sport & Exercise Sciences Research Institute, University of Ulster, Newtownabbey, Northern Ireland; 3 South Eastern Health and Social Care Trust, Belfast, Northern Ireland; 4 Psychology Research Institute, University of Ulster, Co. Londonderry, Northern Ireland; Tehran University of Medical Sciences, Iran (Republic of Islamic)

## Abstract

**Purpose:**

Physical activity (PA) typically declines throughout pregnancy. Low levels of PA are associated with excessive weight gain and subsequently increase risk of pre-eclampsia, gestational diabetes mellitus, hypertension disorders, delivery by caesarean section and stillbirth. Systematic reviews on PA during pregnancy have not explored the efficacy of behaviour change techniques or related theory in altering PA behaviour. This systematic review evaluated the content of PA interventions to reduce the decline of PA in pregnant women with a specific emphasis on the behaviour change techniques employed to elicit this change.

**Search and Review Methodology:**

Literature searches were conducted in eight databases. Strict inclusion and exclusion criteria were employed. Two reviewers independently evaluated each intervention using the behaviour change techniques (BCT) taxonomy to identify the specific behaviour change techniques employed. Two reviewers independently assessed the risk of bias using the guidelines from the Cochrane Collaboration. Overall quality was determined using the GRADE approach.

**Findings:**

A total of 1140 potentially eligible papers were identified from which 14 studies were selected for inclusion. Interventions included counselling (n = 6), structured exercise (n = 6) and education (n = 2). Common behaviour change techniques employed in these studies were goal setting and planning, feedback, repetition and substitution, shaping knowledge and comparison of behaviours. Regular face-to-face meetings were also commonly employed. PA change over time in intervention groups ranged from increases of 28% to decreases of 25%. In 8 out of 10 studies, which provided adequate data, participants in the intervention group were more physically active post intervention than controls.

**Conclusions and Implications:**

Physical activity interventions incorporating behaviour change techniques help reduce the decline in PA throughout pregnancy. Range of behaviour change techniques can be implemented to reduce this decline including goals and planning, shaping knowledge and comparison of outcomes. A lack of high quality interventions hampers conclusions of intervention effectiveness.

## Introduction

Within the European Union, over half of the adult population are classed as being overweight or obese according to their body mass index (BMI ≥25) [1]. Maternal obesity increases health risks for mother and baby such as pre-eclampsia, gestational diabetes mellitus, hypertension disorders, delivery by caesarean section and stillbirth [2], [3]. These risks are present for women who are obese at the time they conceive but increase as women gain weight during pregnancy. Maternal weight gain can persist onto subsequent pregnancies and is positively correlated with adverse risks [4].

### Physical Activity in Pregnancy

For healthy pregnant women, physical activity (PA) is a safe and effective way of reducing adverse health risks. PA can be defined as any bodily movement produced by skeletal muscles that require energy expenditure [5]. This can include activity across a range of domains including, leisure, sports, occupation and domestic [6]. Government agencies across the world, including Canada, and the USA, recommend that all pregnant women should engage in PA throughout their pregnancy [7], [8], [9]. In the absence of UK specific guidelines, healthy pregnant women are advised to undertake 150 minutes or more of moderate intensity PA per week [10], [11].

Physical activity can improve the pregnancy experience for women and the health of their infants and children. Previous studies have found that physically active women report improved physical stamina and mood as well as reduced rates of nausea, fatigue and stress [8], [11], [12], [13]. Women and babies have also experienced long-term benefits stemming from an active pregnancy including active lifestyles and reduced obesity rates [14], [15]. Evidence suggests that pregnancy provides an opportunity to promote positive health behaviours. This opportunity has been branded as a ‘teachable moment’ in a woman’s life, as perceptions of personal risk are increased [16]. In addition, strong emotional responses and a re-definition of their social role and responsibility occurs as a result, pregnant women tend to be more motivated to adopt positive health behaviours, such as physical activity [16], [17]. Despite these benefits and the apparent opportunities offered by pregnancy, PA is often lower in pregnant women than in the general population. Cross sectional population studies using self-report measures of physical activity across the UK and USA estimate, only 3–15% of pregnant women were meeting current guidelines compared with 24–26% of non-pregnant women [18]–[22].

### Behaviour Change Interventions

The identification of the optimal behaviour change techniques (BCTs) necessary for increasing PA have been a topic of considerable research attention [23]. Six important techniques have been identified, these include; providing information on the likely consequences of specific behaviour, action planning, reinforcing effort or progress, providing instructions, facilitative social comparison and time management [23]. The relative value of each of these techniques is likely to depend on the population in question and successful BCTs may differ for a pregnant woman compared to a non-pregnant woman since this is a unique time in her life when she may be more receptive to health promotion [24].

There are many interventions aimed at promoting healthy lifestyles throughout pregnancy. Many of these are multidimensional, incorporating a combination of lifestyle factors. PA is often a secondary outcome of such interventions, as such it receives limited attention. Less focus on PA as an outcome within interventions is evident in existing reviews, which mainly use medical or obstetric outcomes such as gestational weight gain (GWG), gestational diabetes mellitus or preeclampsia. Reviews of lifestyle interventions, specifically for GWG, are varied and report reduced GWG, [25], [26], [27] inconclusive results [28], and no effect [29]. Moreover, these reviews often ignore a consideration of the specific behaviour change techniques employed or relevant theories underlying the intervention. This makes it difficult for researchers and clinicians to understand the key, transferable, intervention components, therefore preventing generalisation of findings within this particular setting. Interventions and their components often have a theoretical basis as a means of understanding, predicting and explaining targeted behaviour change. Social cognitive theories have provided the basis for many of the physical activity interventions reported in the literature. Recognising the determinants of behaviours as well explaining how these determinants influence subsequent behaviour is fundamental to intervention development and implementation [30]. The MRC emphasise that complex interventions should be grounded in theory as theory is important for improving likelihood of effectiveness [31], [32]. Hence it is clear that studies should employ a relevant theory in intervention development which will help pave the way for the selection of appropriate and relevant BCTs.

### Aim of Review

To date, reviews of PA interventions during pregnancy have not investigated how the use of BCTs may contribute to a reduction in the decline in PA with advancing pregnancy. Therefore, this review was undertaken to evaluate the content of PA interventions to reduce the decline of PA in pregnant women with a specific emphasis on the behaviour change techniques that were employed to elicit this change.

### Objectives

To identify the BCT categories incorporated within PA interventions for pregnant women, to assess any change in PA levels between group’s pre and post intervention and, to assess the methodological quality of existing interventions for PA behaviour change during pregnancy.

### Methods

The Preferred Reporting Items for Systematic reviews and Meta-Analysis (PRISMA) Guidelines and Cochrane Systematic Review Methodology, incorporating risk of bias and strength of recommendations were used as a methodological template for this review [33], [34].

### Eligibility Criteria

The review was restricted to randomised controlled trials (RCTs) [33], with application of the following criteria: pregnant women with no known medical or obstetric conditions with BMIs in the range of normal, overweight and obese (BMIs ≥19); interventions designed to maintain or increase PA during pregnancy; and inclusion of PA during pregnancy as an outcome measure. Trials were excluded if they met any of the following criteria: only abstract available as sufficient data were required to identify intervention components and PA measure; inclusion of pregnant women with diagnosed type 1 or type 2 diabetes at recruitment; and interventions specifically designed for underweight pregnant women.

### Search Strategy

Literature searches were conducted between February and May 2012 using the following databases; EMBASE (1980–2012), Medline (1946–2012), AMED (1985–2012), PsycInfo (1806–2012), SportDiscus (1984–2012), CINAHL (1934–2012), PEDro (1929–2012), Cochrane CENTRAL library. All databases were searched from inception to ensure that this kind of review had not been published previously. Current trials or unpublished/grey literature were searched including Index to Thesis, DART Europe, ClinicalTrials.gov and the National Institute for Health Research. Hand searches of relevant journals were performed as well as citation searches using Web of Knowledge (1972–2012). The following search terms were used in different combinations; “pregnancy, pregnant, pregnant women, expectant mothers, prenatal care, prenatal, physical activity, exercise, leisure activities, activities of daily living, human activities, walking, group exercise, physical fitness, aerobic exercises, aquatic exercises, swimming, motor activities, exercise therapy, randomised controlled trial, intervention trials, clinical trials.”

### Study Selection and Data Extraction

The articles were screened by their titles and abstracts. Studies that did not meet the inclusion criteria were excluded. The research team (SC, MS, DL, MM) independently reviewed papers which were ambiguous regarding inclusion. Where disagreements occurred, they were resolved through discussion with all four team members. In the case of duplicate studies the most relevant or most recent was included. Authors of papers were contacted for further information of methodology and data if their study met inclusion criteria but did not give enough detail. In the case of protocols that met the inclusion criteria, the authors were contacted in order to attain any relevant data or recent papers in press.

### Physical Activity Change

Where data were available from published papers and authors, a percentage change in PA within and between groups was calculated. This produced an amount of activity in each study for the intervention and control group. In accordance with recommended guidelines from across the world [7], [8], [9] healthy pregnant women should be engaging in 150 minutes of moderate intensity activity per week. Physical activity can also be expressed in METS or multiples of an individuals resting metabolic rate. MET scores represent the metabolic equivalent intensity levels for activities. Moderate intensity activity is classified as 3–5 METs. Therefore 150 minutes of moderate intensity activity is equivalent to 450–750 MET/minutes per week or 7.5–12.5 MET hours per week [35], [36], [37].

A desirable outcome for intervention effectiveness was classed as an intervention group engaging in greater PA than control group at follow-up, regardless of adherence to guidelines. An undesirable outcome was classed as the intervention group engaging in less PA than controls at follow-up.

Control groups were classified as a comparator intervention or usual care if stated. Usual care involves the standard care provided to all pregnant women.

### Behaviour Change Techniques

All intervention procedures were read in detail and coded using the BCT taxonomy a tool which aims to standardise BCT labels and meanings (version 1.1 September 2012) [38], [39]. The taxonomy facilitates standardisation of techniques across studies to enhance reliably and validity. This tool allows interventions to be classified and compared across disciplines and takes account of interchangeable terms such support and planning. Three authors coded each intervention independently, using the BCT taxonomy and compared results. Where disputes occurred, a fourth member of the team was available.

### Psychological Theories

All interventions were assessed to detect any explicit or implicit mention of relevant social cognitive theories.

### Risk of Bias and Strength of Recommendations

The risk of bias of each study was assessed using the Cochrane methodology [33]. Data were extracted on a pre-designed data extraction form. Two researchers independently assessed risk of bias (SC, DL). Results were compared and a consensus reached for each study. A third member of the team was available if consensus was not reached. The quality of evidence from included trials was also evaluated using Grading Recommendations Assessment, Development and Evaluation (GRADE) [40]. Table 1 details the key constructs used along with risk of bias to determine the quality of evidence.

**Table 1:** Definitions for quality assessment^1^ (external validity).

Construct	Definition
Consistency	Consistency of effect across studies- any unexpected inconsistencies?
	Did the studies show a general trend in behaviour change?
	Did the studies with behaviour change have a common BCT?
Directness	How similar are the participants in the studies and do they relate to a normal pregnant population; Demographics? Comorbidities affecting PA?
Relevance to practice	How feasible to implement these interventions in normal practice? Resources? Patient and health professional time?
Drop-out	Drop-out rates of participants: are rates common to other PA interventions? 20% or less dropout is seen as acceptable for short term interventions (3 months duration or less) and 30% or less drop out is seen as acceptable for long term interventions (greater than 3 months) [32].
Outcome measurement	PA measurement consistency across studies?
Intervention content	Does intervention take into account published guidelines?
	Does intervention addressed give participants definition of any of the four physical activity components: frequency, intensity, type, time?
	Theoretically based interventions?
	How closely do techniques fit with said theory?

1 Adapted from GRADE [40].

## Results

### Study Selection

**Figure 1:**
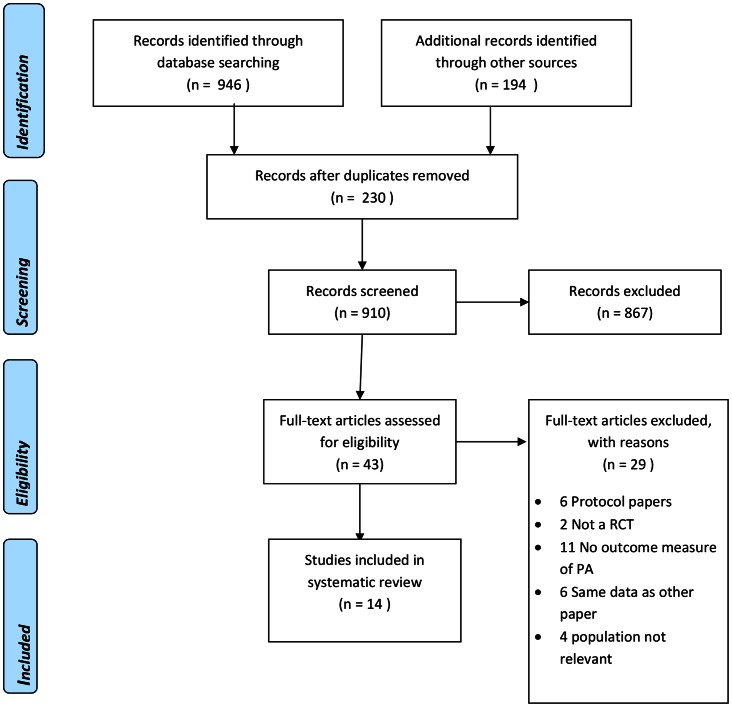
PRISMA flow diagram of literature search for physical activity interventions in pregnancy. The PRISMA flow diagram depicts the flow of information throughout the different phases of this systematic review. It includes the number of records identified, included and excluded and the reasons for exclusions.

Overall, 1140 articles were identified through database, citation and hand searching, 230 duplicates were removed (see flowchart, Figure 1). A total of 14 studies were selected for inclusion.

### Characteristics of Included Studies and Interventions

Table 2 provides a detailed description of the characteristics of included studies. This review contains 14 studies including a total of n = 2553 pregnant women. The overall mean age across the studies was 29.0 (2.0) years. Participant involvement in studies and interventions ranged from one hour to 12 months. The healthcare professional delivering the intervention varied across studies and included nurses, dieticians, physiotherapists, exercise specialists and researchers (Table 3).

**Table 2:** Characteristics of included studies.

Study	Participants	Intervention	PA outcome measure	PA Follow-up times	Effect on PA from baseline to follow-up		Adherence to PA guidelines^1^ at follow-up		
					% change control	% change intervention	Control	Intervention	
Polley et al 2002 [54]	n = 120 pregnant womenunder 20 weeks gestation at recruitment	Written and oral information. Women who exceeded weight recommendations received additional individualised counselling	Paffenbarger exercise questionnaire (Energy expenditure per week)	baseline, 30 weeks gestation and 6 weeks postpartum	Data not supplied	Data not supplied	Data not supplied	Data not supplied	
Rankin 2002 [46]	n = 157 pregnant womenunder 12 weeks gestation at recruitment	Exercise class twice a week. Encouraged to engage in other PA activities as well including aqua-natal classes, home based video.	Self-report diaries	8–12 weeks, 12–16 weeks, 36–40 weeks, 12–16 weeks postpartum	Data not supplied	Data not supplied	Data not supplied	Data not supplied	
Shen et al 2006 [50]	N = 244 pregnant women under 26 weeks gestation at recruitment	Weekly exercise session Exercise video designed for use at home.	Activity questionnaire (Data categorised into unfit, active or fit)	20- 26 weeks and 2 months post enrolment (28–34 weeks)	−10.49	22.52	Cannot categorise	Cannot categorise	
Gaston and Prapavessis 2009 [42]	n = 208 pregnant womenunder 31 weeks gestation at recruitment	one off booklet incorporating 4 main components of PMT.	Godin leisure time exercise questionnaire (MET minutes per week)	baseline, 1 weeks post intervention: differing gestation (14–30 weeks)	−10.24	15.53	No	Yes	
Ong et al 2009∧ [44]	n = 12 obese pregnant women 18 weeks gestation at recruitment	Home based supervised exercise.	Pregnancy physical activity questionnaire (MET hours per week)	18 and 28 weeks	2.46	0.66	No	no	
Yeo 2009 [47]	n = 124 pregnant women less than 14 weeks gestation at recruitment	Walking.	Weekly exercise log	weekly	Data not supplied	Data not supplied	Data not supplied	Data not supplied	
Callaway et al 2010 [41]	n = 50 obese pregnant women at or under 12 weeks gestation at recruitment	individualised exercise plan, encouraged goal setting. Reviews every 4 week with phone calls in between visits.	Pregnancy physical activity questionnaire (MET hours per week)	12, 20, 28, 36 weeks gestation	−58.2	−1	No	yes	
Guelinckx et al2010 [49]	n = 195 obese pregnant women under 15 weeks gestation at recruitment	Counselled in 3 group sessions. Information brochure provided	Baecke questionnaire (likert scale range)	Once during each trimester	−8.36	−4.42	Cannot categorise	Cannot categorise	
Chasan-Taber et al 2011 [48]	n = 208 pregnant sedentary women at risk of gestational diabetes millitus	4 sessions of stage matched counselling with mailed support correspondence	Pregnancy physical activity questionnaire (MET hours per week)	10–12 weeks, 22–24 weeks, 34–34 weeks	−18.90	1.99	Yes	Yes	
Haakstad and Bo 2011 [43]	n = 105 pregnant women under 24 weeks gestation at recruitment	Supervised sessions of aerobic dance exercises.	questions designed and validated by authors	12–24 weeks, 36–38 weeks 6–12 weeks postpartum	Data not supplied	Data not supplied	Data not supplied	Data not supplied	
Huang et al 2011 [51]	n = 240 women between 16 weeks gestation to 6 months postpartum at recruitment	6 one-to-one counselling sessions. Received brochure with information on weight management.	Health promoting lifestyle profile (Walker). Frequency of PA	baseline (16 weeks), 6 months postpartum	3.09	28.76	Cannot categorise	Cannot categorise	
Jackson et al 2011 [52]	n = 327 pregnant womenunder 26 weeks gestation at recruitment	Computer counselling programme	Two questions assessing frequency and duration of PA per week. (minutes per week)	baseline and follow-up at least 4 weeks after intervention (differing gestations)	11.48	22.05	No	Yes	
Luoto et al 2011 [53]	n = 442 pregnant women between 8–12 weeks gestation at recruitment	4 PA counselling sessions and 3 diet counselling sessions. Monthly group exercise.	Measure by Aitasillo et al. (MET per week)	8–12 weeks, 26–28 weeks, 36–37 weeks	−35.63	−33.72	Yes	Yes	
Oostdam et al 2012 [45]	n = 121 pregnant women at risk of GDM under 20 weeks gestation at recruitment	60 minute Supervised exercise programme 2 days a week during.	Accelerometer (Counts per week)	15, 24 and 31 weeks	−18.37	−25.35	Yes	Yes	

1 In accordance with published UK guidelines [10].

∧ Author provided PA data which was not in published paper.

MET: Metabolic equivalent.

**Table 3:** Intervention components and BCTs employed.^1.^

Study	Delivery	Approx. duration of intervention^2^	Intervention classification	BCT categories	Outcome
Polley et al 2002 [54]	Masters and doctorial level staff trained in nutrition or clinical psychology	26 weeks (not explicitly stated)	Generic Education and individualised counselling	Feedback and monitoring	Not known
				Associations	
				Goals and Planning	
Rankin 2002 [46]	Researcher	32 weeks (not explicitly stated)	Structured exercise, generic	Shaping knowledge	Not known
				Repetition and substitution	
				Comparison of behaviour	
				Feedback and monitoring	
				Antecedents	
Shen et al 2006[50]	Licenced fitness trainers	10–16 weeks	Structured exercise, generic	Feedback and monitoring	Desirable
				Repetition and substitution	
				Shaping Knowledge	
				Comparison of behaviour	
				antecedents	
Gaston and Prapavessis 2009 [42]	None- read brochure alone	1 week (given brochure and follow-up taken 1 week after)	Educational/counselling, generic	Goals and Planning	Desirable
				Natural consequences	
				Shaping knowledge	
Ong et al 2009 [44]	Not stated	10 weeks	Structured exercise, generic	Repetition and substitution	undesirable
				Antecedent	
Yeo 2009 [47]	Exercise specialist	22 weeks (not explicitly stated)	Structured exercise, generic	Repetition and substitution	Not known
				Shaping Knowledge	
				Feedback and monitoring	
				Antecedents	
				Reward and threat	
Callaway et al 2010 [41]	Exercise physiologist and dietician	24 weeks (not explicitly stated)	Counselling, individualised	Goals and Planning	Desirable
				Feedback and monitoring	
				Social Support	
Guelinckx et al 2010 [49]	Trained nutritionist	22 weeks (not explicitly stated)	Educational		Desirable
Chasan-Taber et al 2011 [48]	Health educator	12 weeks	Counselling	Feedback and monitoring	Desirable
				Repetition and substitution	
				Goals and planning	
				Comparison of outcomes	
Haakstad and Bo 2011 [43]	Certified aerobics instructor	12 weeks	Structured exercise, generic	Shaping knowledge	Not known
				Repetition and substitution	
				Goals and Planning	
				Comparison of behaviour	
Huang et al 2011 [51]	Masters-prepared nurse with training in nutrition and PA	12 months (pregnant at baseline subjects only)	Counselling, individualised	Feedback and monitoring	Desirable
				Goals and Planning	
Jackson et al 2011 [52]	Video	One pre-appointment computer programme (duration not explicitly stated)	Educational		Desirable
Luoto et al 2011 [53]	Nurse	29 weeks	Counselling, individualised	Comparison of behaviour	desirable
				Goals and Planning	
				Repetition and substitution	
				Shaping Knowledge	
Oostdam et al 2012 [45]	Trained physiotherapist	25 weeks (not explicitly stated)	Structured exercise, generic	Repetition and substitution	Undesirable
				Shaping knowledge	
				Comparison of behaviour	

1 Categorised using BCT taxonomy [38], [39].

2 Time during which intervention was being administered.

There were a variety of focus behaviours across studies including exercise/physical activity [41]–[48], diet [49] or both diet and PA [50]–[54]. Only two papers [42], [48] explicitly employed a theoretically based intervention using the protection motivation theory (PMT) and a combination of the Transtheoretical Model and Social Cognitive Theory respectively [55], [56], [57].

Twelve studies described their control groups as receiving standard care. There was no clear definition in these papers of standard care. The two studies with no usual care group compared their intervention with a stretching group [47] or a health and wellness group [48]. The stretching group were provided with a 40 minute videotape instructing slow muscle movements which did not contain aerobic or muscle resistance components. The health and wellness group received face-to-face sessions with a health educator to discuss general issues related to health and wellness during pregnancy with information which is readily available to the public.

### Effect of Intervention on PA Behaviour

There were a wide variety of measures used to assess PA throughout the included papers (Table 2).

Percentage PA behaviour change over time between groups is detailed in table 2. Given that PA tends to decrease progressively through pregnancy, any outcome that demonstrates greater PA than controls is deemed to be a desirable outcome. This was indeed the case in 8 of the 10 papers providing PA data. Physical activity levels increased more in the intervention groups compared with control groups in 5 studies [42], [48], [50]–[52] and decreased less than controls in 3 studies [41], [49], [53]. Two studies demonstrated an undesirable effect where PA was higher in the control group at follow-up compared with the intervention group [44], [45]. A sign and binomial test indicated that the chance of observing 8 or more desirable outcomes in 10 trials has a one-tailed p-value of 0.0547.

Of the 7 studies providing PA data measured through minutes per week or METs, three reported the intervention group met PA guidelines at follow up but this was not achieved in the control groups [41], [42], [52]. Three studies demonstrated adherence to PA guidelines by both groups at follow-up [45], [48], [53].

From eight interventions reporting a desirable behaviour change between groups at follow up, 5 involved regular face-to-face meetings [41], [48], [50], [51], [53], three were offered over a 20 week period [41], [49], [53], and four focused mainly on PA [41], [42], [48], [51] as opposed to diet only or a combination of diet and PA.

### Behaviour Change Techniques

Table 3 details standardisation of intervention components using the BCT taxonomy [38], [39].

**Figure 2:**
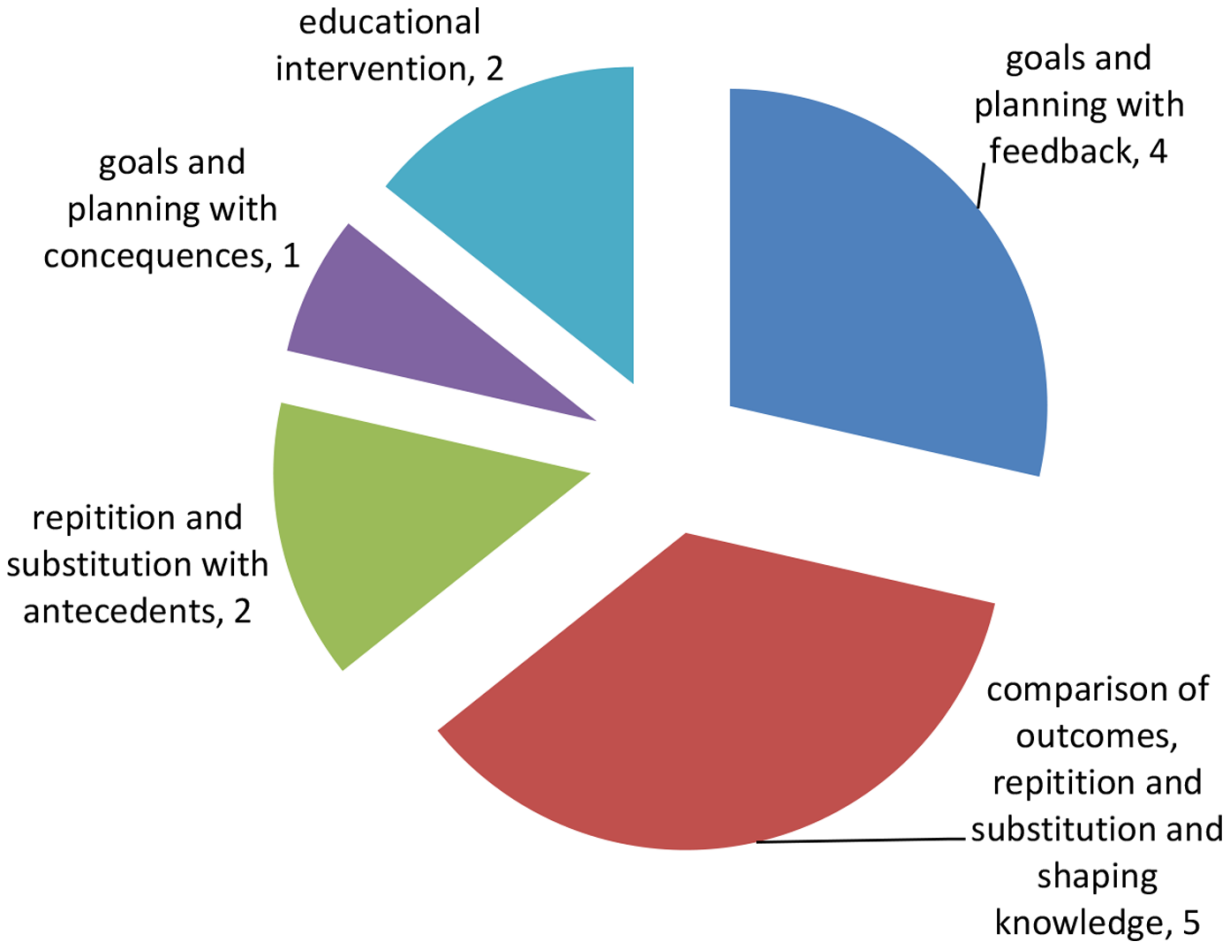
Breakdown of BCT combinations in physical activity interventions (n = 14). The pie chart depicts the number of studies which employ specific BCT combinations.

Interventions often consisted of a range of behaviour change techniques and four main approaches were used (Figure 2):

Goals and Planning with feedback. All classed as individualised counselling by authors [41], [48], [51], [54].

Comparison of outcomes, repetition and substitution and shaping knowledge [43], [45], [46], [50], [53]. Four classed as structured exercise programme by author and one counselling [53].

Repetition and substitution with antecedents [44], [47].

Goals and planning with natural consequences [42].

Two studies contained no BCTs according to the BCT taxonomy as they were educational interventions and simply provided information to participants [49], [52].

### PA Behaviour Change and BCTs

PA behaviour ranged in each of the combinations of techniques:

Goals and planning with feedback: Three studies implementing these techniques demonstrated desirable PA behaviour change [41], [48], [51] Furthermore, all four studies indicated significantly higher PA levels at follow-up in the intervention group compared with control group.

Comparison of outcomes, repetition and substitution and shaping knowledge: two of the five studies implementing comparison of outcomes, repetition and substitution and shaping knowledge, demonstrated desirable PA behaviour change [50], [53]. One had undesirable PA outcomes with greater PA in the control group compared with intervention group at follow-up [45] and the others did not provide the PA data [43], [46].

Repetition and substitution with antecedents: One study produced undesirable PA behaviour change [44] and the other did not provide adequate data to analyse [47].

Goals and planning with natural consequences: Gaston et al found a desirable PA behaviour change. [42].

The two educational interventions demonstrated desirable PA behaviour change [49], [52].

### Psychological Theories

Two papers used an explicit theoretical approach - protection motivation theory (PMT) [52], [55], Transtheoretical Model and Social Cognitive Theory [48], [56], [57]. Each of the theoretical components were well represented and described in both interventions. The remaining studies used a range of components including motivational, behavioural-enaction and stage theories.

### Risk of Bias

Table 4 shows the outcome of the assessment of risk of bias within studies.

**Table 4:** Risk of Bias assessment within studies.

Study	Random sequence generation	Allocation concealment	Blinding of participants and personnel	Blinding of outcome assessment	Incompleteoutcome data	Selectivereporting	Other bias
Polley et al 2002 [54]	UNCLEAR	UNCLEAR	HIGH	HIGH	LOW	UNCLEAR	UNCLEAR
Rankin 2002 [46]	LOW	LOW	HIGH	HIGH	LOW	LOW	LOW
Shen et al 2006 [50]	LOW	LOW	HIGH	HIGH	LOW	HIGH	HIGH
Gaston and Prapavessis 2009 [42]	LOW	HIGH	LOW	LOW	UNCLEAR	LOW	LOW
Ong et al 2009 [44]	UNCLEAR	LOW	HIGH	UNCLEAR	HIGH	UNCLEAR	HIGH
Yeo 2009 [47]	LOW	LOW	HIGH	LOW	UNCLEAR	LOW	HIGH
Callaway et al 2010 [41]	LOW	UNCLEAR	HIGH	HIGH	LOW	LOW	HIGH
Guelinckx et al 2010 [49]	UNCLEAR	UNCLEAR	UNCLEAR	UNCLEAR	LOW	LOW	LOW
Chasan-Taber et al 2011 [48]	UNCLEAR	UNCLEAR	UNCLEAR	UNCLEAR	LOW	LOW	LOW
Haakstad and Bo 2011 [43]	LOW	UNCLEAR	HIGH	LOW	LOW	LOW	HIGH
Huang et al 2011 [51]	LOW	HIGH	LOW	LOW	LOW	LOW	LOW
Jackson et al 2011 [52]	LOW	LOW	HIGH	HIGH	LOW	HIGH	HIGH
Luoto et al 2011 [53]	LOW	UNCLEAR	HIGH	HIGH	LOW	LOW	UNCLEAR
Oostdam et al 2012 [45]	LOW	UNCLEAR	HIGH	LOW	LOW	LOW	HIGH

#### Low risk of bias

Selection bias was low in 10 of the included studies. Random sequence generation had been performed robustly and described clearly in these papers. Attrition bias was similarly low in 11 of the included studies, as was reporting bias with 10 indicating low risk of bias.

#### High risk of bias

Failure to blind participants to group allocation as well as failure to blind involved personnel resulted in high risk of performance bias in 10 of the included studies. Furthermore, as many participants were not blinded, the use of self-report measures introduced further detection bias in 7 studies. Seven studies scored highly for other risks of bias. Common reasons for this were participant data differing between groups at baseline, no reporting of demographics such as age, education or parity and no reporting of any co-interventions.

#### Unclear risk of bias

Risk of bias was unclear for at least one study in all 7 domains. Studies tended to be classed as unclear in response to poor reporting of design, process or outcomes. Seven of the 14 studies were classed as unclear for risk of selection bias. These studies did not give a clear indication of how allocation was performed or concealed.

### Quality

Table 1 provides details of the operational definitions of external validity used in this review.

#### Consistency

Of the ten studies that provided data, eight indicated that the intervention group engaged in greater PA than controls at follow-up. The direction of change in behaviour varied across studies. For intervention groups, 6 studies indicated increases in PA and 4 indicated decreases in PA over time. There were some common BCTs such as goals and planning and repetition and substitution.

#### Directness

Participant age range was 25–34 years across studies. Parity varied between the studies with two recruiting first time mothers only [42], [45] and others including all nulliparous and multiparous women. Most studies included a range of BMIs, but two included obese participants only. Characteristics regarding ethnicity, education and marital status were not consistently reported but where information was provided, there was a range in all domains. These studies allow for generalizability to the pregnant population aged between 25 and 34 years.

#### Relevance to practice

Intervention delivery personnel varied (see table 3). In most studies external personnel were brought in to implement the intervention. Personnel had to be trained specifically to deliver the intervention in three studies, which adds a significant time and cost of an intervention. Location of intervention delivery was mostly in the hospital setting where participants normally received treatment. Other locations included the participant’s home and community centres. These studies can be integrated into practice, but would require training of staff and funding for their roles.

#### Drop-out rates

Drop out rates ranged from zero to 43%. Interventions with a longer duration tended to have greater drop-out rates. Three studies demonstrated acceptable drop-out rates within the recommended rates of 20% and lower for short term interventions (3 months or less) and 30% or lower for long term interventions (over 3 months) [33], [51]–[53]. Six of the other studies which provided data, had drop-out rates higher than recommended [43], [45], [46], [48], [49], [54].

#### Outcome measures

A wide range of assessment tools for PA were used and only one study had an objective measure [45]. The most common measure for PA was MET score, derived from self-report activity. Included studies tended to produce an average MET hours/minutes per week/day. The range of scale and categorical outcome measures reduces external validity due to limited comparability across studies.

#### Intervention content

Four studies used current PA guidelines as the basis for intervention design and delivery [43], [46], [48], [53]. Furthermore 7 studies described the four components of physical activity (frequency, intensity, time and type) [43]–[47], [49]. Of the other studies, none of these components were described. Only two interventions were explicitly designed around a behaviour change theory [42], [48].

Overall the quality of evidence was classed as low due to heterogeneity of PA measure, high risk of detection bias and poor reporting of data and processes.

## Discussion

### Summary and Interpretation of Findings

This systematic review suggests that interventions focusing on PA behaviour can reduce the decline or increase PA during pregnancy. A range of individual BCTs can be employed to achieve this outcome but the most effective of these appears to be goals and planning with feedback. Despite wide variations in duration, delivery, behavioural focus and design, the review suggests that interventions with regular face-to-face meetings were more likely to produce positive changes in physical activity behaviour.

One of the common BCTs employed in the interventions included in this review was individualised goal setting and planning. Implementation of such a technique is common in PA interventions for other populations. A recent review found that such action planning was a fundamental technique for increasing PA behaviours in the general population [23]. Furthermore, personalising goals and planning is consistent with evidence from NICE guidelines for PA improvement which suggests that primary care practitioners should use a person-centred approach, recognising an individual’s needs and motivations and agreeing goals with them [58]. A recent meta-analysis assessing the influence of BCT’s within lifestyle interventions (diet and physical activity) to reduce gestational weight gain also found that setting behavioural goals was a common technique in interventions. However, these authors found this BCT to be evident in both effective and ineffective interventions. These findings may be influenced by the inclusion of both diet and PA interventions in analysis [27].

The two educational interventions included in this review [49], [52] indicated positive PA outcomes as a result education from a trained nutritionist and a video doctor, respectively. The intervention groups in these studies engaged in greater PA than controls at follow-up. Unfortunately these interventions did not involve techniques from the BCT taxonomy. Educational interventions involving information provision alone are thought to influence intention rather than behaviour and it is therefore interesting that behaviour was positively affected in the pregnant women involved in these studies [59]. Given that pregnancy is often seen as an opportunity for health promotion [16], education from health professionals may have helped transfer intention into behaviour in these two studies.

The wide range of BCTs employed suggests that a wide range of combinations of techniques may be beneficial when trying to alter physical activity behaviour in pregnant women. Although no one specific technique can be recommended the outcome of this review provides health professionals with options for helping pregnant women remain active. By emphasising personal goals and planning, providing information and demonstrating behaviours or delivering education about the importance of PA and what can be done.

Despite the MRC guidelines, which suggest that complex interventions should be based upon theory [31], only two of the 14 studies used theory to guide the development of their intervention. Theoretically driven interventions allow generalizability of the findings, and an understanding of the mechanisms from construct to behaviour [60]. Furthermore, Brown et al [61] have identified that theory driven interventions are more likely to address the psychological needs of the individual as well as behaviour change. The application of the MRC guidelines for complex interventions in future studies would offer greater insight into why interventions did or did not work allowing future studies to use this information to inform theory based intervention design [32].

### Methodological Quality

Overall the quality of evidence in the studies included in this review was classed as low. This was due to limitations in study design and reporting, in particular, blinding of personnel. As most interventions were delivered by a person, it would be difficult to blind the personnel delivering the intervention. Blinding of participants was also poorly implemented. As there was no attentional control group in most studies, the participants were aware which group they were in. In addition, the use of self-report measures and non-blinding of participants provides a challenge to physical activity research and increase risk of bias. Social desirability may result in women in the intervention groups exaggerating their self-reported physical activity in order to please the research of intervention facilitators. For this reason the increasing availability of low-cost, objective measures of physical activity is recommended in future research as these can add further validity and allow for more and accurate recording of data [61].

Further issues across studies included poor reporting of data, and differences at baseline between groups. Participant differences in physical activity at baseline have the potential to influence the outcome to a greater extent than the intervention itself. Since pregnancy is seen as a time of natural decline in physical activity, active individuals often find it easier to maintain or minimise the decline in physical activity throughout pregnancy, and pre-pregnancy PA is a consistent predictor of PA through pregnancy [62], [63]. An additional theme across studies was the lack of author definitions of acceptable drop-out rates. Cochrane recognise that 20% or less dropout is acceptable for short term interventions (3 months duration or less) and 30% or less drop out is acceptable for long term interventions (greater than 3 months) [33]. Although the drop-out rates were similar to those reported in existing PA in pregnancy reviews [64] which found a range of 10 to 50%, only three studies demonstrated acceptable drop-out rates as detailed by Cochrane [33]. This reduces validity of the studies as interventions may be having a moderation or mediation effect rather than a direct effect on PA behaviour.

The wide range of PA measures used in the studies reviewed presents a problem for researchers and practitioners trying to draw conclusions on the efficacy of different interventions. Although all of the self-report measured were validated, in many instances this validation was not in pregnant women. There was limited information provided about the validation procedure employed. Physical activity research has recognised the importance of objective measurement [65], with accelerometry being the gold standard for measuring PA behaviour. In this review, only one study used accelerometers, again reducing validity of study results.

### Strengths and Limitations

This review takes an original approach to assessing lifestyle interventions for pregnant women by specifically focusing on physical activity behaviour change and demonstrates that decline in activity throughout pregnancy can be reduced or even alleviated through intervention. In addition, use of the latest BCT taxonomy is an originality of this paper, as this new tool gives researchers, practitioners and interdisciplinary teams a shared language and understanding of the intervention process.

The key limitations of this review stem from the inadequate reporting of PA data and poor intervention design. Only two of the six papers which were classed as exercise programmes provided adequate data for analysis. Therefore, this type of intervention and related BCT’s could not be assessed thoroughly in relation to PA behaviour change. PA was often added on as an additional element to nutritional interventions and therefore many studies failed to report PA data, making it difficult to measure the effect of the intervention on behaviour. In addition, PA data were assessed using the last follow-up time in each study thus reducing homogeneity between studies with follow-up ranging from 14 weeks gestation to 12 months postpartum. This is an important factor as current research demonstrates that PA declines naturally as the pregnancy proceeds. This review should assist researchers in the design of future interventions, encouraging them to objectively measure PA and address the evident limitations such as high risk of bias, poor reporting and lack of theoretical basis.

### Conclusions and Implications

Given the importance of PA to many subsequent outcomes, it is important that clinicians have clearer guidance on the most effective behaviour change techniques which can be used to enhance and sustain PA behaviour throughout pregnancy. Based on the results of the current review, it is possible that women can become or stay physically active throughout their pregnancy. The fundamental component to this activity is implementation of lifestyle interventions. These lifestyle interventions are most effective when behaviour change techniques are employed and delivered face-to-face. Training for midwives and nurses in BCT implementation could enhance delivery of health messages and intervention at routine patient appointments. This review provides new knowledge about the benefits of interventions incorporating behaviour change techniques for increasing PA throughout pregnancy. It is important for those health professionals who are involved in the provision of antenatal education and public health messages.

## Supporting Information

Figure S1 **PRISMA 2009 Checklist.** PRISMA checklist contains 27 checklist items relevant to the content of a systematic review and meta-analysis, which include the title, abstract, methods, results, discussion and funding.
(DOC)
